# Supplementary material for: Self-Assembling Injectable Hydrogel for Controlled Drug Delivery of Antimuscular Atrophy Drug Tilorone
Source: Pharmaceutics. 2022 Dec 6;14(12):2723. doi: 10.3390/pharmaceutics14122723 (PMC9782908; doi:10.3390/pharmaceutics14122723)
Supplement: Supplementary file 1 [file pharmaceutics-14-02723-s001.zip › pharmaceutics-1971684-supplementary.pdf]

Supplementary Materials

Self-Assembling Injectable Hydrogel for Controlled Drug Delivery of Antimuscular Atrophy Drug Tilorone

Mohamed M. Abdelghafour [1,2], Ágota Deák [1], Tamás Kiss [3], Mária Budai-Szűcs [3], Gábor Katona [3], Rita Ambrus [3], Bálint Lőrinczi [4], Anikó Keller-Pintér [5], István Szatmári [4], Diána Szabó [6], László Rovó [6] and László Janovák [1,*]

1 Department of Physical Chemistry and Materials Science, University of Szeged, Rerrich Béla tér 1, H-6720 Szeged, Hungary
2 Department of Chemistry, Faculty of Science, Zagazig University, Zagazig 44519, Egypt
3 Institute of Pharmaceutical Technology and Regulatory Affairs, University of Szeged, Eötvös str. 6., H-6720 Szeged, Hungary
4 Institute of Pharmaceutical Chemistry, University of Szeged, Szeged, Eötvös str. 6, H-6720 Szeged, Hungary
5 Department of Biochemistry, Albert Szent-Györgyi Medical School, University of Szeged, H-6720 Szeged, Hungary
6 Department of Oto-Rhino-Laryngology and Head & Neck Surgery, University of Szeged, Tisza Lajos krt. 111, H-6724 Szeged, Hungary
* Correspondence: janovakl@chem.u-szeged.hu; Tel.: +36-62-544-210; Fax: +36-62-544-042

Methods

Gel permeation chromatography with a refractive index detector (Shimadzu RID-10A, Japan) was used to determine the molecular weight and molecular weight distribution in terms of polydispersity of chitosan and PVA. Two linear columns (Ultrahydrogel, 7.8×300 mm, Waters) were utilized, and the eluting solvent was acetate buffer solution at pH 5.0 at a flow rate of 0.8 mL/min at 35 °C for chitosan, while the aqueous solution of 0.1 M sodium nitrate and 0.02% (w/v) sodium azide with a flow rate of 1mL/min at 35 °C was used in the case of PVA. Various molecular weight standards of poly(ethylene glycol) were used to calibrate the GPC procedure. As it can be seen the natural Chitosan macromolecule has wider molecular weight distribution (PI=3.12) than the syntetic PVA (PI=2.01).

Results

Table S1. The polydispersity index of the initial PVA and initial Chitosan.

Sample	Mn (g/mol)	Mw (g/mol)	Polydispersity (Mw/Mn)
Chitosan	27,400	85,600	3.12
PVA	23,300	46,800	2.01

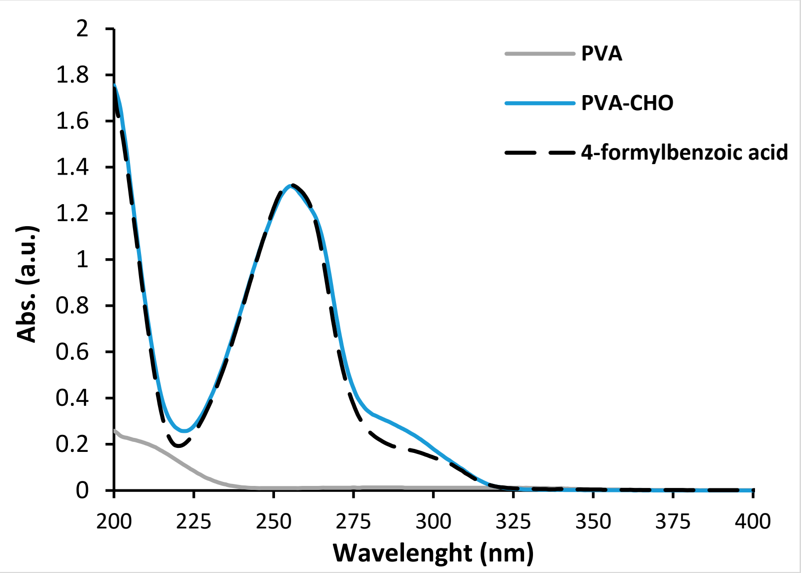

Figure S1. UV absorbance spectra of PVA, PVA-CHO, and 4-formylbenzoic acid.

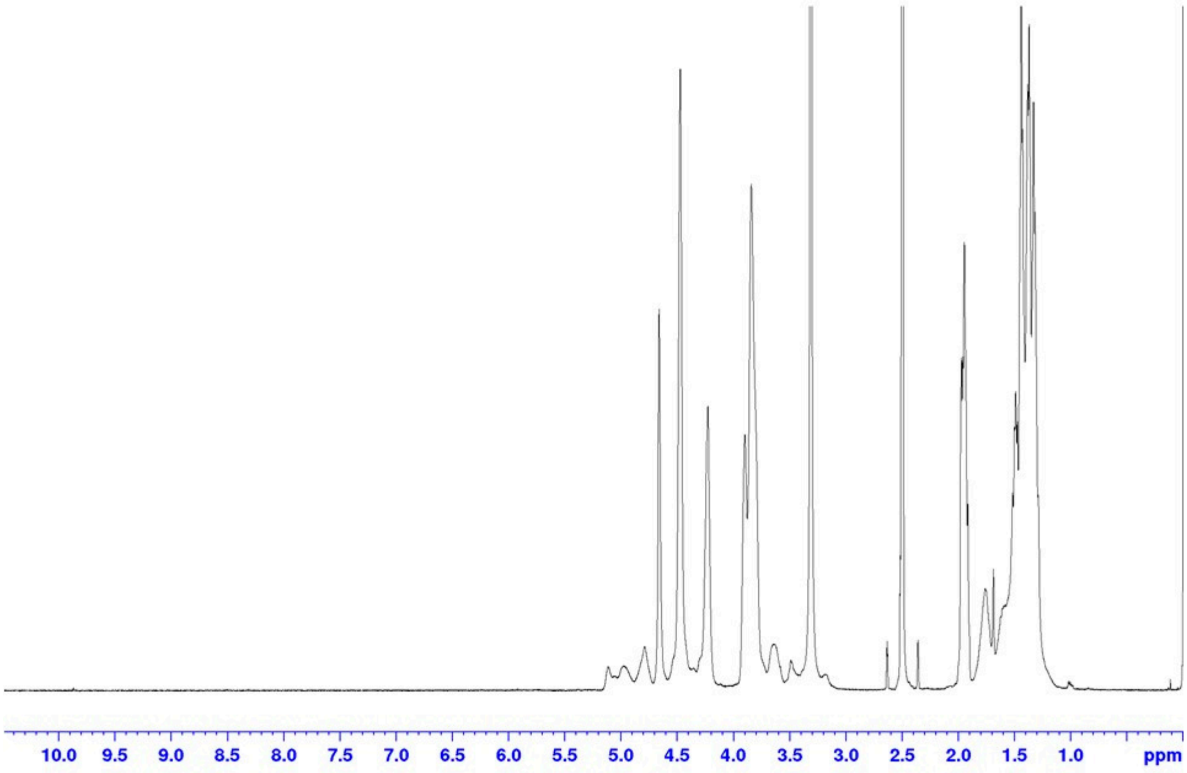

Figure S2. ^{1}H-NMR spectrum of **PVA.**

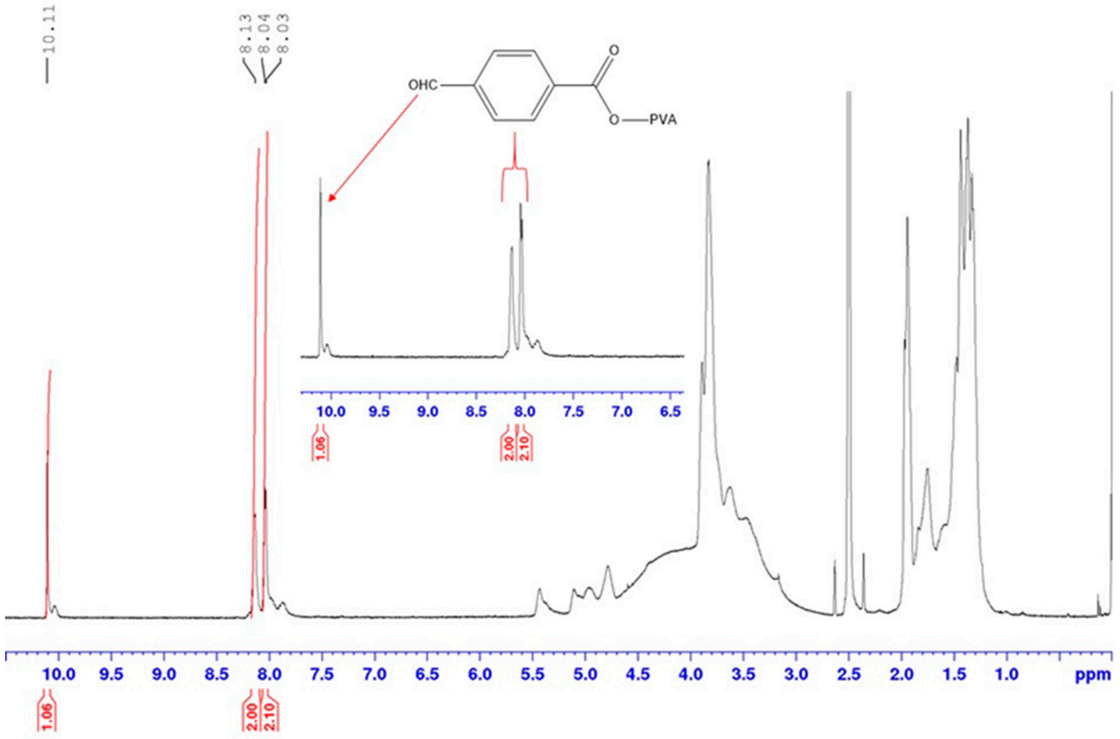

Figure S3. ^{1}H-NMR spectrum of **PVA-CHO**.

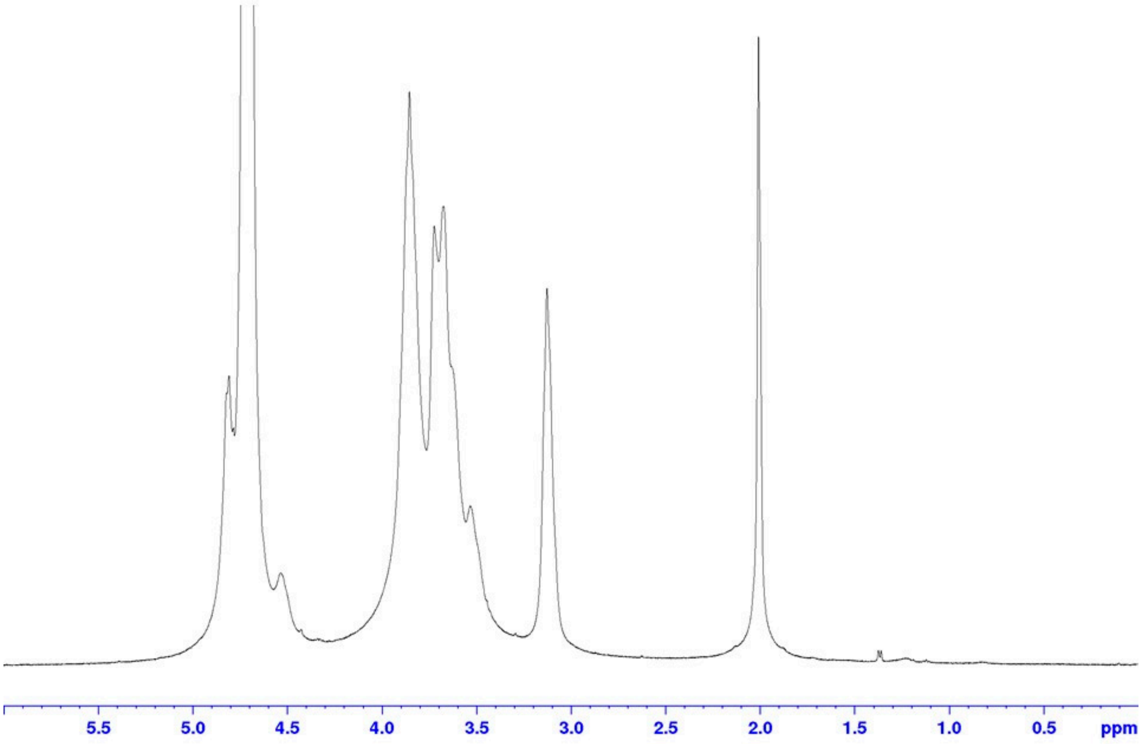

Figure S4. ^{1}H-NMR spectrum of **Chitosan**.

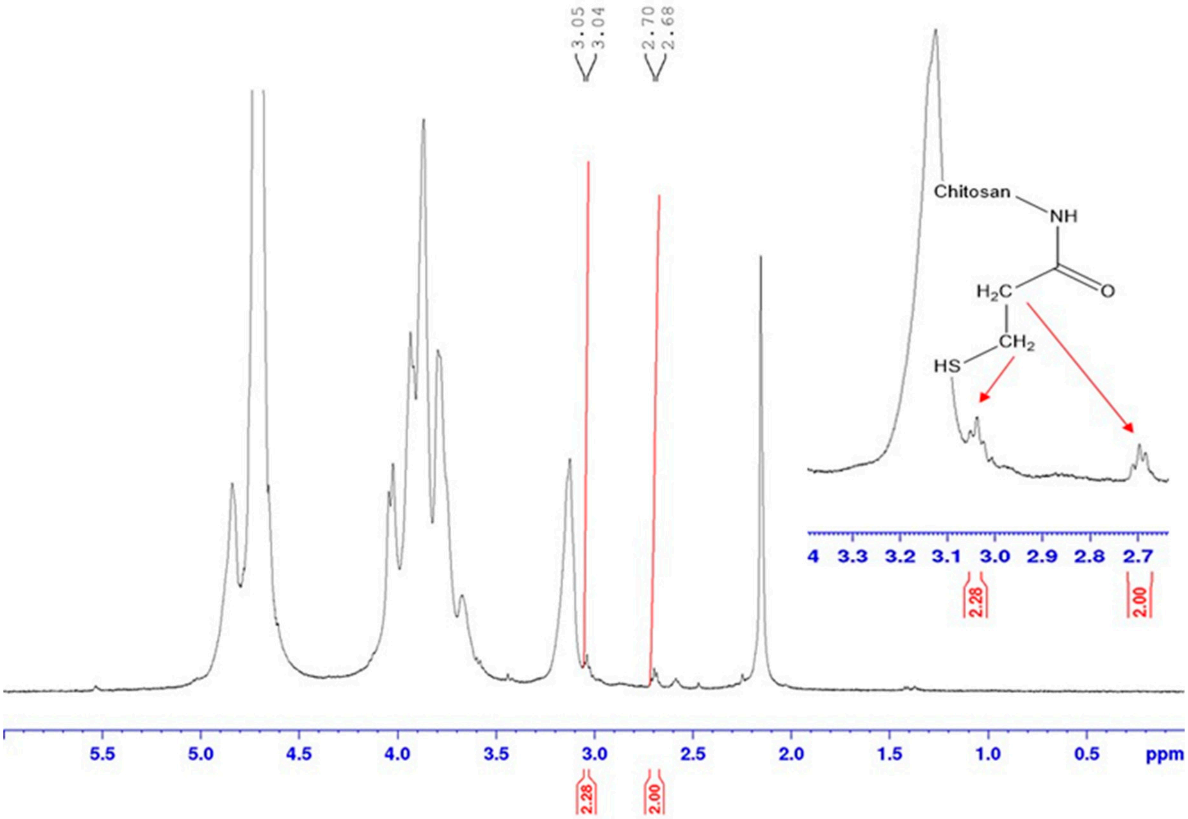

Figure S5. ^{1}H-NMR spectrum of **CHIT-SH.**

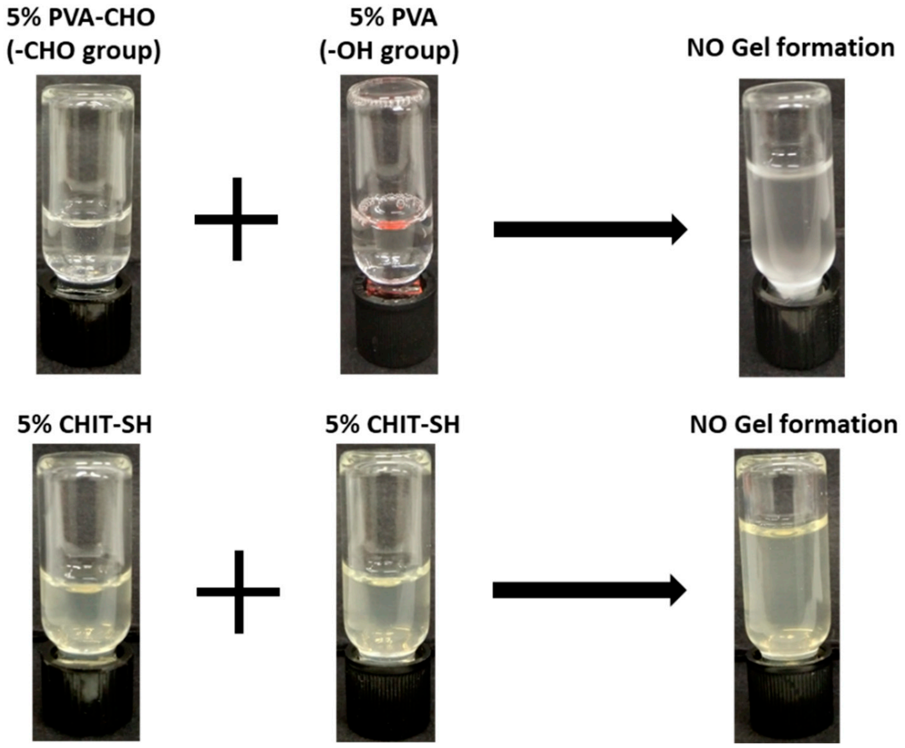

Figure S6. The possible bond formation in the hydrogel system.

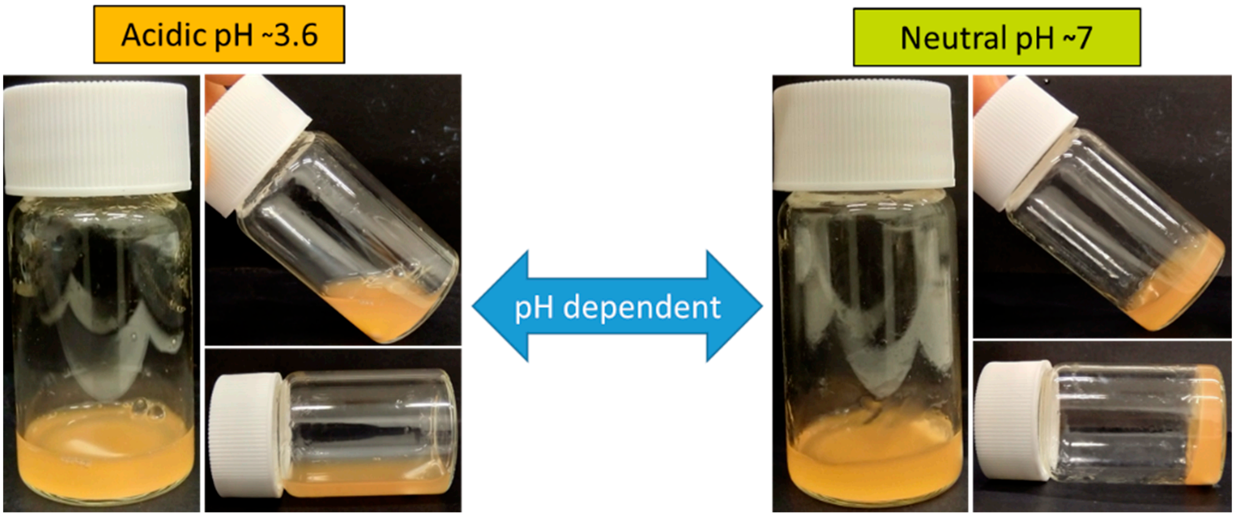

Figure S7. Photos of the PVA-CHIT-Tilorone pH-dependent hydrogel (2%w/v polymer concentration with 0.023 mg/mL Tilorone content).

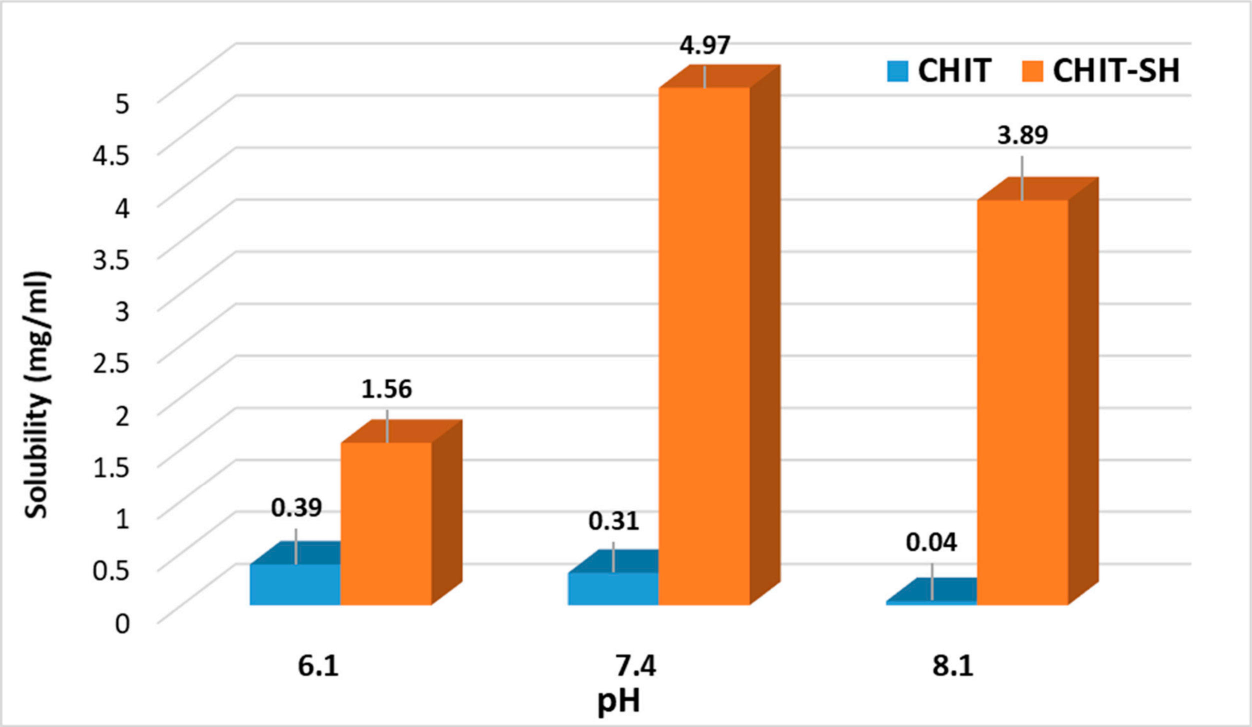

Figure S8. The solubility of chitosan and modified chitosan in different pH buffer solutions.

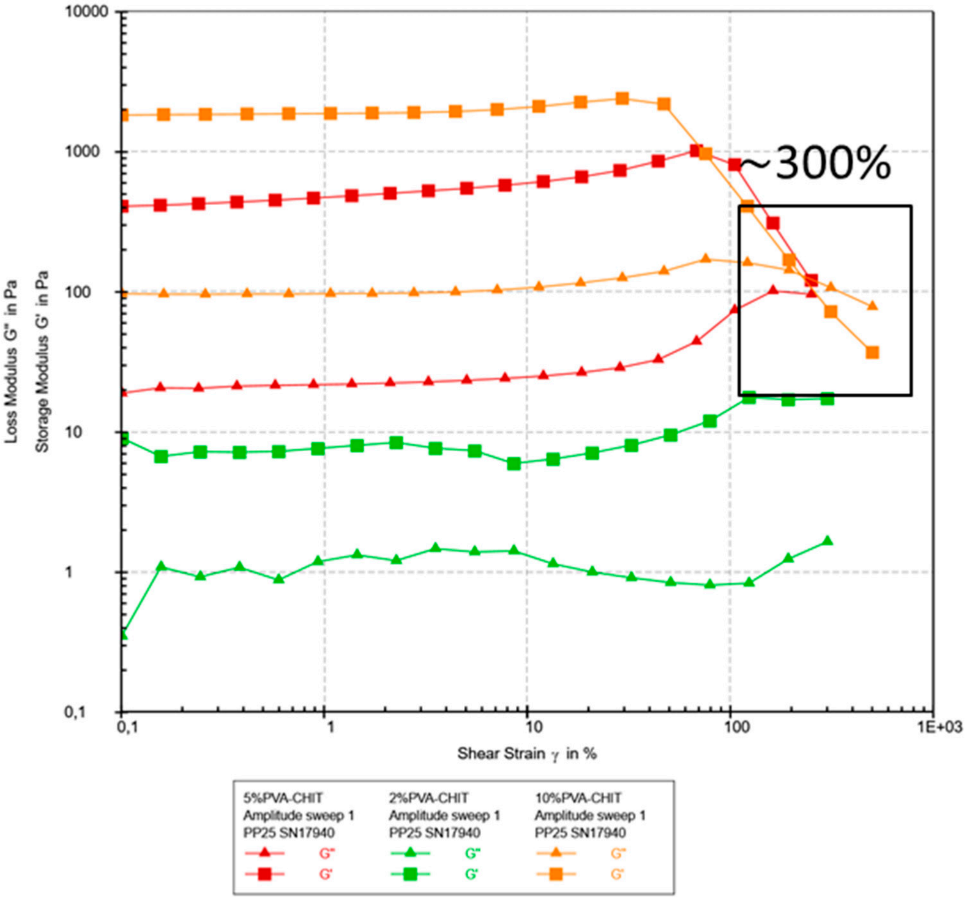

Figure S9. Amplitude sweep with the elastic modulus and viscous modulus at different shear strains.

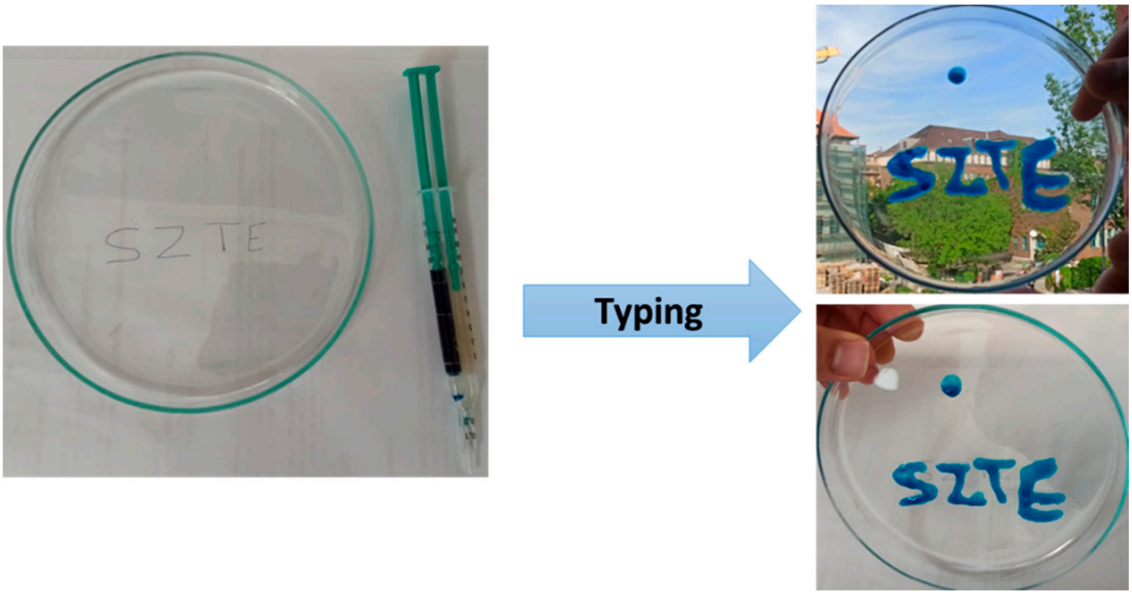

Figure S10. Photos of the static mixing tool between two solutions of PVA-CHO (2%w/v) and Chitosan-SH (2%w/v) to print the abbreviation name of the University of Szeged.

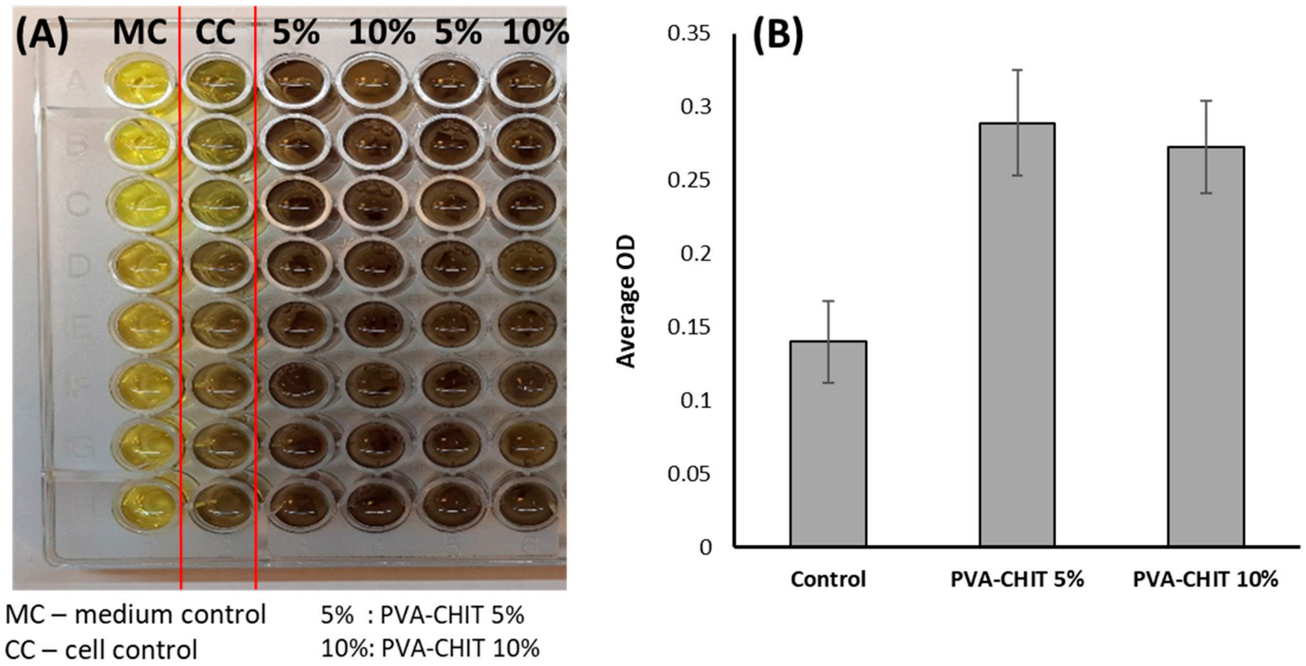

Figure S11. Biocompatibility of hydrogel; (**A**) The photo of the cell viability of MRC-5 for PVA-CHIT 5% and PVA-CHIT 10% and compared with cell control, (**B**) Average OD for PVA-CHIT 5% and PVA-CHIT 10% and compared with cell control.
